# Tris(piperazinediium) phosphatododeca­molybo(V,VI)phosphate

**DOI:** 10.1107/S1600536810002473

**Published:** 2010-02-06

**Authors:** Yu-kun Lu, Ji-qing Xu, Hai-hui Yu

**Affiliations:** a College of Chemistry and Chemical Engineering, China University of Petroleum (East China), Qingdao Shandong 266555, People’s Republic of China; b College of Chemistry and State Key Laboratory of Inorganic Synthesis and Preparative Chemistry, Jilin University, Changchun 130023, People’s Republic of China; c College of Chemical Engineering, Northeast Dianli University, Jilin 132012, People’s Republic of China

## Abstract

The title compound, (C_4_H_12_N_2_)_3_[PMo_12_O_40_] or  (H_2_pip)_3_[PMo_12_O_40_] (pip is piperazine), was prepared under hydro­thermal conditions. The asymmetric unit contains one-sixth of a mixed-valent Mo(V,VI) pseudo-Keggin-type [PMo_12_O_40_]^6−^ anion and half a piperazinediium cation, (H_2_pip)^2+^. The discrete Keggin-type [PMo_12_O_40_]^6- ^anion has 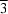 site symmetry and the three (H_2_pip)^2+^ cations each have 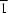 site symmetry at the centres of the mol­ecules. The central P atom is on special position 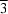, which is a roto-inversion position and generates the disorder of the PO_4_ tetra­hedron. Furthermore, six doubly bridging oxide groups are also disordered with an occupancy factor of 0.5 for each O atom. The anions and cations are linked by an extensive network of inter­molecular N—H⋯O and C—H⋯O hydrogen bonds.

## Related literature

For polyoxometalate chemistry, see: Pope & Müller (1991 ▶); Hill (1998 ▶); Kurth *et al.* (2001 ▶). For related structures, see: Han *et al.* (2005 ▶); Li *et al.* (2007 ▶); Yuan *et al.* (2008 ▶). For general background to bond-valence calculations, see: Brown & Altermatt (1985 ▶).
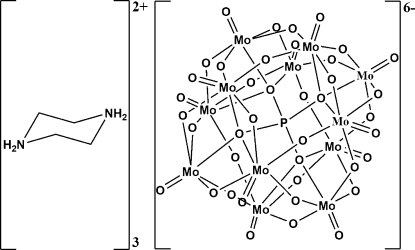


## Experimental

### 

#### Crystal data

(C_4_H_12_N_2_)_3_[PMo_12_O_40_]
*M* *_r_* = 2086.72
Trigonal, 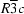

*a* = 17.890 (3) Å
*c* = 23.600 (6) Å
*V* = 6541 (2) Å^3^
*Z* = 6
Mo *K*α radiation
μ = 3.49 mm^−1^
*T* = 296 K
0.20 × 0.16 × 0.11 mm

#### Data collection

Rigaku R-AXIS RAPID diffractometer
Absorption correction: multi-scan (*ABSCOR*; Higashi, 1995 ▶) *T*_min_ = 0.517, *T*_max_ = 0.682
11291 measured reflections
1413 independent reflections
1363 reflections with *I* > 2σ(*I*)
*R*_int_ = 0.056

#### Refinement

*R*[*F* ^2^ > 2σ(*F* ^2^)] = 0.020
*wR*(*F* ^2^) = 0.053
*S* = 1.14
1413 reflections
142 parameters
H-atom parameters constrained
Δρ_max_ = 0.66 e Å^−3^
Δρ_min_ = −0.61 e Å^−3^

### 

Data collection: *RAPID-AUTO* (Rigaku, 1998 ▶); cell refinement: *RAPID-AUTO*; data reduction: *RAPID-AUTO*; program(s) used to solve structure: *SHELXS97* (Sheldrick, 2008 ▶); program(s) used to refine structure: *SHELXL97* (Sheldrick, 2008 ▶); molecular graphics: *DIAMOND* (Brandenburg, 1999 ▶); software used to prepare material for publication: *SHELXL97*.

## Supplementary Material

Crystal structure: contains datablocks I, global. DOI: 10.1107/S1600536810002473/si2235sup1.cif

Structure factors: contains datablocks I. DOI: 10.1107/S1600536810002473/si2235Isup2.hkl

Additional supplementary materials:  crystallographic information; 3D view; checkCIF report

## Figures and Tables

**Table 1:** Hydrogen-bond geometry (Å, °)

*D*—H⋯*A*	*D*—H	H⋯*A*	*D*⋯*A*	*D*—H⋯*A*
N1—H1*C*⋯O6	0.90	2.22	2.812 (4)	123
N1—H1*D*⋯O50	0.90	2.43	2.926 (6)	115
N1—H1*C*⋯O4^i^	0.90	2.20	3.041 (7)	155
N1—H1*C*⋯O40^i^	0.90	2.16	3.047 (7)	168
N1—H1*C*⋯O30^ii^	0.90	2.52	3.091 (6)	122
N1—H1*D*⋯O5	0.90	2.19	2.852 (6)	130
N1—H1*D*⋯O1^iii^	0.90	2.14	2.900 (4)	142
C1—H1*A*⋯O6	0.97	2.58	3.101 (5)	114
C1—H1*B*⋯O4^iv^	0.97	2.58	3.347 (7)	137
C2—H2*A*⋯O2^iv^	0.97	2.43	3.291 (4)	148
C2—H2*B*⋯O3^ii^	0.97	2.43	3.156 (6)	132
C2—H2*B*⋯O2^v^	0.97	2.42	3.068 (4)	124

Symmetry codes: (i) 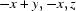; (ii) 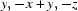; (iii) 
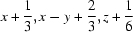
; (iv) 
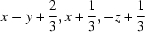
; (v) 
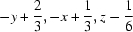
.

**Fig. 1.**
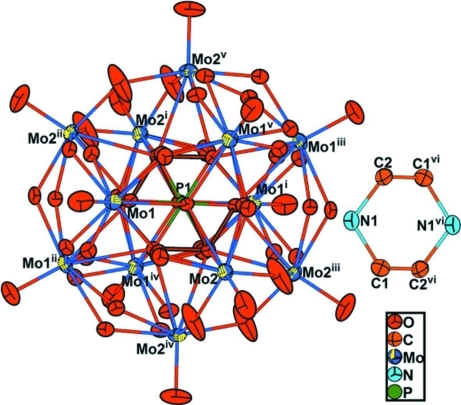
A view of the molecule of (I) with displacement ellipsoids drawn at the 30% probability level. H atoms have been omitted. [Symmetry codes: (i) -x, -y, -z; (ii)x-y, x, -z; (iii) -x + y, -x, z; (iv) -y, x-y, z; (v) y, -x + y, -z; (vi) -x + 2/3,-y + 1/3, -z + 1/3]

**Fig. 2.**
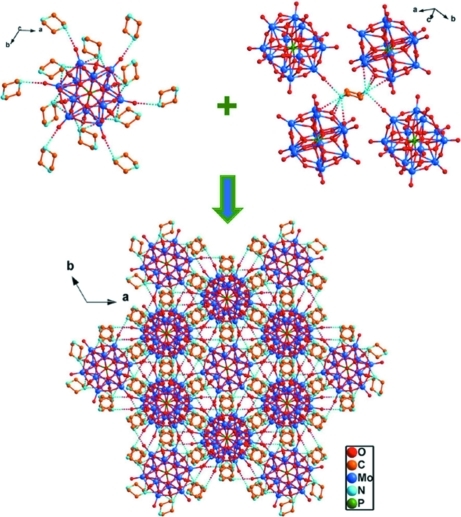
Ball-stick representation of the three-dimensional supramolecular network structure of (I).

## supplementary crystallographic information

### Comment

Polyoxometalates (POMs) comprise a rich and diverse family of metal oxygen clusters made up of early transition metals (primarily including W, Mo and V) with unique redox, acidic, magnetic and catalytic properties (Pope & Müller, 1991; Hill, 1998). The Keggin-type structure was of epoch-making significance in the history of POMs chemistry (Kurth, 2001). The Keggin-type polyanions, [PMo_12_O_40_]^3-^, have been indicated as excellent building blocks to construct novel compounds (Li *et al.*, 2007; Yuan *et al.*, 2008).

The structure of the title compound consists of a discrete polyoxoanion [PMo_12_O_40_]^6-^ and three diprotonated piperazine molecules. The heteropolyoxoanion [PMo_12_O_40_]^6-^ has a roto-inversion symmetry with the P1 atom located on the 3 centre. The pseudo-Keggin unit [PMo_12_O_40_]^6-^ may be viewed as a shell of {Mo_12_O_36_} encapsulating a disordered {PO_4_} moiety, present at its center and responsible for the local tetrahedral geometry. The central P atom is surrounded by a cube of eight oxygen (six O7 and two O8) atoms with each of them half occupied due to the inversion symmetry at P1, and each oxygen of the {PO_4_} group covalently bonded to three different molybdenum centers of the shell (Fig. 1). All Mo centers possess similar distorted octahedral geometry MoO_6_ defined by one terminal oxygen atom, four doubly bridging oxo-groups and one central oxygen atom. Six doubly bridging oxo-groups (O3, O30, O4, O40, O5 and O50) are disordered with occupancy factor 0.5.

Extensive hydrogen bonding interactions help to stabilize the structure (Table 1). Each (H_2_pip)^2+ ^cation donates eight N—H···O hydrogen bonds to eight bridging oxygen atoms from two [PMo_12_O_40_]^6-^ anions and two ones to two terminal oxygen atoms from the other two [PMo_12_O_40_]^6- ^anions. Each [PMo_12_O_40_]^6-^ anion joins twelve (H_2_pip)^2+ ^cations to generate a three-dimensional supramolecular network structure (Fig.2).

Result of bond valence sum (Brown & Altermatt, 1985) calculation for Mo centers gives the average value 5.71 (5.52 for Mo1 and 5.89 for Mo2) in good agreement with the expected value of 5.75, which reveals that there exist three Mo^V^ and nine Mo^VI^ atoms in the Keggin-type compound. The three classes of Mo—O average distances are 1.663, 1.925 and 2.482 Å, being obviously larger than the corresponding distances in [PMo_12_O_40_]^5-^ (1.638, 1.891 and 2.443 Å) (Han *et al.*, 2005).

### Experimental

A mixture of KH_2_PO_4_.2H_2_O(0.70 g, 5 mmol), MoO_3_.2H_2_O (0.45 g, 2.5 mmol), pip (0.43 g, 5 mmol), H_3_BO_3 _(0.31 g, 5 mmol) and 18 ml water was stirred for 2 h in air; it was adjusted to pH = 1 with HCl solution (18 wt %) and was heated in a 25 ml stainless steel reactor with a Teflon-liner at 180°C for 5 days, and then cooled to room temperature. Black polyhedron crystals were isolated with 71% yield (based on Mo). Elemental analysis for 1: Anal. Calcd: C, 6.91; H, 1.74; N, 4.03; found: C, 6.96; H, 1.67; N, 4.11.

### Refinement

All H atoms were placed at calculated positions (H–C = 0.97 Å), with *U*_iso_(H) = 1.2 *U*_eq_(C) and (H–N = 0.90 Å), with *U*_iso_(H) = 1.2*U*_eq_(N).

### Crystal data

**Table:**

(C_4_H_12_N_2_)_3_[PMo_12_O_40_]	*D*_x_ = 3.178 Mg m^−^^3^
*M**_r_* = 2086.72	Mo *K*α radiation, λ = 0.71073 Å
Trigonal, *R*3*c*	Cell parameters from 7741 reflections
*a* = 17.890 (3) Å	θ = 2.3–25.9°
*c* = 23.600 (6) Å	µ = 3.49 mm^−^^1^
*V* = 6541 (2) Å^3^	*T* = 296 K
*Z* = 6	Polyhedron, black
*F*(000) = 5934	0.20 × 0.16 × 0.11 mm

### Data collection

**Table:**

Rigaku R-AXIS RAPID diffractometer	1413 independent reflections
Radiation source: fine-focus sealed tube	1363 reflections with *I* > 2σ(*I*)
graphite	*R*_int_ = 0.056
Detector resolution: 10 pixels mm^-1^	θ_max_ = 25.9°, θ_min_ = 2.2°
ω scans	*h* = −21→20
Absorption correction: multi-scan (*ABSCOR*; Higashi, 1995)	*k* = −21→21
*T*_min_ = 0.517, *T*_max_ = 0.682	*l* = −25→28
11291 measured reflections	

### Refinement

**Table:**

Refinement on *F*^2^	Secondary atom site location: difference Fourier map
Least-squares matrix: full	Hydrogen site location: inferred from neighbouring sites
*R*[*F*^2^ > 2σ(*F*^2^)] = 0.020	H-atom parameters constrained
*wR*(*F*^2^) = 0.053	*w* = 1/[σ^2^(*F*_o_^2^) + (0.0243*P*)^2^ + 29.698*P*] where *P* = (*F*_o_^2^ + 2*F*_c_^2^)/3
*S* = 1.14	(Δ/σ)_max_ < 0.001
1413 reflections	Δρ_max_ = 0.66 e Å^−^^3^
142 parameters	Δρ_min_ = −0.61 e Å^−^^3^
0 restraints	Extinction correction: *SHELXL97* (Sheldrick, 2008), Fc^*^=kFc[1+0.001xFc^2^λ^3^/sin(2θ)]^-1/4^
Primary atom site location: structure-invariant direct methods	Extinction coefficient: 0.000246 (16)

### Special details

**Table:**

Geometry. All e.s.d.'s (except the e.s.d. in the dihedral angle between two l.s. planes) are estimated using the full covariance matrix. The cell e.s.d.'s are taken into account individually in the estimation of e.s.d.'s in distances, angles and torsion angles; correlations between e.s.d.'s in cell parameters are only used when they are defined by crystal symmetry. An approximate (isotropic) treatment of cell e.s.d.'s is used for estimating e.s.d.'s involving l.s. planes.
Refinement. Refinement of *F*^2^ against ALL reflections. The weighted *R*-factor *wR* and goodness of fit *S* are based on *F*^2^, conventional *R*-factors *R* are based on *F*, with *F* set to zero for negative *F*^2^. The threshold expression of *F*^2^ > σ(*F*^2^) is used only for calculating *R*-factors(gt) *etc*. and is not relevant to the choice of reflections for refinement. *R*-factors based on *F*^2^ are statistically about twice as large as those based on *F*, and *R*- factors based on ALL data will be even larger.

### Fractional atomic coordinates and isotropic or equivalent isotropic displacement parameters (Å^2^)

**Table:**

	*x*	*y*	*z*	*U*_iso_*/*U*_eq_	Occ. (<1)
O40	−0.0237 (5)	0.1531 (4)	0.0737 (3)	0.0207 (13)	0.50
O30	0.0735 (4)	0.2139 (4)	−0.0190 (2)	0.0197 (11)	0.50
O50	0.1366 (4)	0.1818 (4)	0.0714 (2)	0.0194 (11)	0.50
C1	0.2710 (2)	0.1678 (2)	0.20485 (14)	0.0309 (8)	
H1A	0.2122	0.1414	0.2187	0.037*	
H1B	0.3058	0.2205	0.2261	0.037*	
C2	0.3615 (2)	0.2263 (2)	0.11974 (13)	0.0268 (7)	
H2A	0.3992	0.2809	0.1383	0.032*	
H2B	0.3605	0.2373	0.0796	0.032*	
Mo1	−0.038697 (18)	0.177638 (17)	0.000676 (10)	0.02000 (11)	
Mo2	0.046862 (16)	0.131369 (17)	0.123257 (11)	0.01944 (11)	
N1	0.27315 (17)	0.18861 (18)	0.14324 (12)	0.0247 (6)	
H1C	0.2375	0.1402	0.1241	0.030*	
H1D	0.2541	0.2262	0.1386	0.030*	
O1	−0.05695 (18)	0.26048 (17)	0.00042 (11)	0.0377 (6)	
O2	0.06793 (17)	0.1903 (2)	0.18145 (11)	0.0451 (7)	
O3	0.0872 (4)	0.2510 (4)	−0.0075 (2)	0.0184 (11)	0.50
O4	−0.0138 (4)	0.1855 (4)	0.0855 (3)	0.0191 (13)	0.50
O5	0.1516 (4)	0.2187 (4)	0.0842 (2)	0.0187 (11)	0.50
O6	0.09680 (17)	0.06339 (16)	0.14146 (18)	0.0583 (10)	
O7	0.0325 (2)	0.0921 (2)	0.02241 (16)	0.0151 (8)	0.50
O8	0.0000	0.0000	0.0639 (3)	0.0148 (13)	0.50
P1	0.0000	0.0000	0.0000	0.0119 (3)	

### Atomic displacement parameters (Å^2^)

**Table:**

	*U*^11^	*U*^22^	*U*^33^	*U*^12^	*U*^13^	*U*^23^
O40	0.027 (3)	0.026 (4)	0.017 (3)	0.019 (3)	−0.001 (2)	−0.003 (2)
O30	0.019 (3)	0.020 (3)	0.020 (3)	0.010 (3)	0.000 (2)	0.000 (2)
O50	0.019 (3)	0.021 (3)	0.018 (3)	0.010 (3)	0.000 (2)	−0.002 (2)
C1	0.0290 (18)	0.045 (2)	0.0244 (16)	0.0228 (16)	0.0041 (13)	0.0029 (14)
C2	0.0221 (16)	0.0303 (17)	0.0239 (16)	0.0099 (14)	−0.0001 (12)	0.0070 (13)
Mo1	0.02413 (17)	0.01744 (16)	0.02263 (17)	0.01355 (12)	0.00507 (10)	0.00335 (9)
Mo2	0.01881 (16)	0.02439 (17)	0.01642 (16)	0.01178 (12)	−0.00270 (9)	−0.00659 (10)
N1	0.0208 (13)	0.0267 (14)	0.0283 (14)	0.0131 (11)	−0.0023 (11)	0.0028 (11)
O1	0.0555 (17)	0.0341 (14)	0.0390 (14)	0.0340 (13)	0.0132 (12)	0.0100 (11)
O2	0.0340 (15)	0.0623 (19)	0.0436 (16)	0.0276 (14)	−0.0132 (12)	−0.0350 (14)
O3	0.020 (3)	0.014 (3)	0.022 (3)	0.009 (3)	−0.001 (2)	0.001 (2)
O4	0.023 (3)	0.020 (3)	0.021 (3)	0.015 (3)	0.000 (2)	−0.005 (2)
O5	0.021 (3)	0.019 (3)	0.018 (3)	0.011 (3)	−0.001 (2)	−0.003 (2)
O6	0.0199 (13)	0.0171 (13)	0.137 (3)	0.0088 (10)	0.0052 (16)	0.0017 (15)
O7	0.0169 (19)	0.016 (2)	0.0123 (18)	0.0078 (16)	0.0009 (15)	0.0017 (14)
O8	0.015 (2)	0.015 (2)	0.015 (3)	0.0074 (10)	0.000	0.000
P1	0.0117 (5)	0.0117 (5)	0.0124 (8)	0.0058 (2)	0.000	0.000

### Geometric parameters (Å, °)

**Table:**

O40—O4	0.586 (5)	Mo2—O6	1.884 (3)
O40—Mo1	1.830 (7)	Mo2—O6^iv^	1.921 (3)
O40—Mo2	1.898 (7)	Mo2—O5	1.968 (6)
O30—O3	0.641 (5)	Mo2—O4	1.991 (7)
O30—Mo1	1.833 (6)	Mo2—O7	2.458 (4)
O30—Mo1^i^	1.892 (6)	Mo2—O8	2.494 (4)
O50—O5	0.650 (5)	N1—H1C	0.9000
O50—Mo1^i^	1.833 (5)	N1—H1D	0.9000
O50—Mo2	1.855 (6)	O3—Mo1^i^	2.007 (6)
C1—N1	1.497 (4)	O5—Mo1^i^	2.061 (5)
C1—C2^ii^	1.504 (5)	O6—Mo2^v^	1.921 (3)
C1—H1A	0.9700	O7—P1	1.541 (4)
C1—H1B	0.9700	O7—O8	1.747 (5)
C2—N1	1.481 (4)	O7—O7^iii^	1.793 (5)
C2—C1^ii^	1.504 (5)	O7—O7^i^	1.793 (5)
C2—H2A	0.9700	O7—Mo1^i^	2.492 (4)
C2—H2B	0.9700	O8—P1	1.508 (7)
Mo1—O1	1.669 (2)	O8—O7^v^	1.747 (5)
Mo1—O50^iii^	1.833 (5)	O8—O7^iv^	1.747 (5)
Mo1—O30^iii^	1.892 (6)	O8—Mo2^v^	2.494 (4)
Mo1—O3	1.970 (6)	O8—Mo2^iv^	2.494 (4)
Mo1—O3^iii^	2.007 (6)	P1—O8^vi^	1.508 (7)
Mo1—O4	2.039 (7)	P1—O7^i^	1.541 (4)
Mo1—O5^iii^	2.061 (5)	P1—O7^vi^	1.541 (4)
Mo1—O7	2.485 (4)	P1—O7^v^	1.541 (4)
Mo1—O7^iii^	2.492 (4)	P1—O7^iii^	1.541 (4)
Mo2—O2	1.656 (2)	P1—O7^iv^	1.541 (4)
			
O4—O40—Mo1	102.6 (13)	O6—Mo2—O7	92.98 (15)
O4—O40—Mo2	90.4 (13)	O40—Mo2—O7	58.4 (2)
Mo1—O40—Mo2	145.2 (4)	O6^iv^—Mo2—O7	92.46 (15)
O3—O30—Mo1	92.6 (9)	O5—Mo2—O7	72.26 (18)
O3—O30—Mo1^i^	90.9 (9)	O4—Mo2—O7	72.59 (19)
Mo1—O30—Mo1^i^	147.3 (3)	O2—Mo2—O8	158.13 (17)
O5—O50—Mo1^i^	101.3 (9)	O50—Mo2—O8	83.6 (2)
O5—O50—Mo2	90.2 (9)	O6—Mo2—O8	64.16 (14)
Mo1^i^—O50—Mo2	151.6 (3)	O40—Mo2—O8	84.4 (2)
N1—C1—C2^ii^	110.1 (3)	O6^iv^—Mo2—O8	63.75 (14)
N1—C1—H1A	109.6	O5—Mo2—O8	102.89 (18)
C2^ii^—C1—H1A	109.6	O4—Mo2—O8	101.46 (19)
N1—C1—H1B	109.6	O7—Mo2—O8	41.32 (15)
C2^ii^—C1—H1B	109.6	C2—N1—C1	111.5 (2)
H1A—C1—H1B	108.1	C2—N1—H1C	109.3
N1—C2—C1^ii^	110.2 (3)	C1—N1—H1C	109.3
N1—C2—H2A	109.6	C2—N1—H1D	109.3
C1^ii^—C2—H2A	109.6	C1—N1—H1D	109.3
N1—C2—H2B	109.6	H1C—N1—H1D	108.0
C1^ii^—C2—H2B	109.6	O30—O3—Mo1	68.4 (9)
H2A—C2—H2B	108.1	O30—O3—Mo1^i^	70.5 (9)
O1—Mo1—O40	109.5 (2)	Mo1—O3—Mo1^i^	128.0 (3)
O1—Mo1—O50^iii^	110.5 (2)	O40—O4—Mo2	72.5 (12)
O40—Mo1—O50^iii^	139.9 (3)	O40—O4—Mo1	61.1 (12)
O1—Mo1—O30	110.5 (2)	Mo2—O4—Mo1	124.0 (3)
O40—Mo1—O30	93.3 (3)	O50—O5—Mo2	70.5 (8)
O50^iii^—Mo1—O30	75.3 (3)	O50—O5—Mo1^i^	60.7 (8)
O1—Mo1—O30^iii^	110.9 (2)	Mo2—O5—Mo1^i^	125.0 (3)
O40—Mo1—O30^iii^	74.5 (3)	Mo2—O6—Mo2^v^	139.8 (2)
O50^iii^—Mo1—O30^iii^	88.9 (2)	P1—O7—O8	54.2 (2)
O30—Mo1—O30^iii^	138.6 (4)	P1—O7—O7^iii^	54.43 (7)
O1—Mo1—O3	94.28 (19)	O8—O7—O7^iii^	89.79 (17)
O40—Mo1—O3	89.8 (3)	P1—O7—O7^i^	54.43 (7)
O50^iii^—Mo1—O3	89.9 (2)	O8—O7—O7^i^	89.79 (17)
O30—Mo1—O3	18.98 (15)	O7^iii^—O7—O7^i^	88.7 (3)
O30^iii^—Mo1—O3	153.5 (3)	P1—O7—Mo2	124.6 (2)
O1—Mo1—O3^iii^	94.68 (19)	O8—O7—Mo2	70.4 (2)
O40—Mo1—O3^iii^	88.2 (3)	O7^iii^—O7—Mo2	132.5 (2)
O50^iii^—Mo1—O3^iii^	86.0 (2)	O7^i^—O7—Mo2	132.1 (2)
O30—Mo1—O3^iii^	152.5 (3)	P1—O7—Mo1	123.5 (2)
O30^iii^—Mo1—O3^iii^	18.62 (15)	O8—O7—Mo1	131.7 (2)
O3—Mo1—O3^iii^	171.0 (3)	O7^iii^—O7—Mo1	69.08 (18)
O1—Mo1—O4	94.32 (19)	O7^i^—O7—Mo1	130.5 (3)
O40—Mo1—O4	16.3 (2)	Mo2—O7—Mo1	92.09 (13)
O50^iii^—Mo1—O4	154.9 (2)	P1—O7—Mo1^i^	123.1 (2)
O30—Mo1—O4	93.5 (3)	O8—O7—Mo1^i^	132.1 (2)
O30^iii^—Mo1—O4	85.0 (3)	O7^iii^—O7—Mo1^i^	129.8 (3)
O3—Mo1—O4	85.0 (3)	O7^i^—O7—Mo1^i^	68.69 (18)
O3^iii^—Mo1—O4	95.3 (3)	Mo2—O7—Mo1^i^	92.48 (13)
O1—Mo1—O5^iii^	93.53 (18)	Mo1—O7—Mo1^i^	91.83 (12)
O40—Mo1—O5^iii^	155.4 (2)	P1—O8—O7^v^	55.9 (2)
O50^iii^—Mo1—O5^iii^	18.00 (17)	P1—O8—O7^iv^	55.9 (2)
O30—Mo1—O5^iii^	86.4 (2)	O7^v^—O8—O7^iv^	91.7 (3)
O30^iii^—Mo1—O5^iii^	89.6 (2)	P1—O8—O7	55.9 (2)
O3—Mo1—O5^iii^	97.2 (2)	O7^v^—O8—O7	91.7 (3)
O3^iii^—Mo1—O5^iii^	81.2 (2)	O7^iv^—O8—O7	91.7 (3)
O4—Mo1—O5^iii^	171.7 (2)	P1—O8—Mo2	124.17 (12)
O1—Mo1—O7	159.29 (14)	O7^v^—O8—Mo2	130.90 (13)
O40—Mo1—O7	58.4 (2)	O7^iv^—O8—Mo2	131.30 (13)
O50^iii^—Mo1—O7	83.9 (2)	O7—O8—Mo2	68.26 (14)
O30—Mo1—O7	57.5 (2)	P1—O8—Mo2^v^	124.17 (12)
O30^iii^—Mo1—O7	83.3 (2)	O7^v^—O8—Mo2^v^	68.26 (14)
O3—Mo1—O7	70.30 (18)	O7^iv^—O8—Mo2^v^	130.90 (13)
O3^iii^—Mo1—O7	101.25 (18)	O7—O8—Mo2^v^	131.30 (13)
O4—Mo1—O7	71.25 (19)	Mo2—O8—Mo2^v^	91.53 (17)
O5^iii^—Mo1—O7	101.87 (18)	P1—O8—Mo2^iv^	124.17 (12)
O1—Mo1—O7^iii^	158.48 (13)	O7^v^—O8—Mo2^iv^	131.30 (13)
O40—Mo1—O7^iii^	85.4 (2)	O7^iv^—O8—Mo2^iv^	68.26 (14)
O50^iii^—Mo1—O7^iii^	55.5 (2)	O7—O8—Mo2^iv^	130.90 (13)
O30—Mo1—O7^iii^	83.1 (2)	Mo2—O8—Mo2^iv^	91.53 (17)
O30^iii^—Mo1—O7^iii^	56.9 (2)	Mo2^v^—O8—Mo2^iv^	91.53 (17)
O3—Mo1—O7^iii^	101.45 (18)	O8^vi^—P1—O8	180.0
O3^iii^—Mo1—O7^iii^	69.63 (18)	O8^vi^—P1—O7^i^	69.92 (14)
O4—Mo1—O7^iii^	101.55 (19)	O8—P1—O7^i^	110.08 (14)
O5^iii^—Mo1—O7^iii^	70.14 (18)	O8^vi^—P1—O7^vi^	69.92 (14)
O7—Mo1—O7^iii^	42.23 (15)	O8—P1—O7^vi^	110.08 (14)
O2—Mo2—O50	111.8 (2)	O7^i^—P1—O7^vi^	108.86 (14)
O2—Mo2—O6	101.02 (16)	O8^vi^—P1—O7	110.08 (14)
O50—Mo2—O6	83.7 (2)	O8—P1—O7	69.92 (14)
O2—Mo2—O40	110.1 (2)	O7^i^—P1—O7	71.14 (14)
O50—Mo2—O40	89.8 (3)	O7^vi^—P1—O7	180.0 (4)
O6—Mo2—O40	148.3 (2)	O8^vi^—P1—O7^v^	110.08 (14)
O2—Mo2—O6^iv^	101.43 (16)	O8—P1—O7^v^	69.92 (14)
O50—Mo2—O6^iv^	146.7 (2)	O7^i^—P1—O7^v^	71.14 (14)
O6—Mo2—O6^iv^	87.82 (16)	O7^vi^—P1—O7^v^	71.14 (14)
O40—Mo2—O6^iv^	80.8 (2)	O7—P1—O7^v^	108.86 (14)
O2—Mo2—O5	93.44 (19)	O8^vi^—P1—O7^iii^	69.92 (14)
O50—Mo2—O5	19.28 (15)	O8—P1—O7^iii^	110.08 (14)
O6—Mo2—O5	92.81 (19)	O7^i^—P1—O7^iii^	108.86 (14)
O40—Mo2—O5	90.9 (3)	O7^vi^—P1—O7^iii^	108.86 (14)
O6^iv^—Mo2—O5	164.7 (2)	O7—P1—O7^iii^	71.14 (14)
O2—Mo2—O4	93.90 (19)	O7^v^—P1—O7^iii^	180.0 (3)
O50—Mo2—O4	91.0 (3)	O8^vi^—P1—O7^iv^	110.08 (14)
O6—Mo2—O4	165.1 (2)	O8—P1—O7^iv^	69.92 (14)
O40—Mo2—O4	17.11 (16)	O7^i^—P1—O7^iv^	180.0 (3)
O6^iv^—Mo2—O4	89.0 (2)	O7^vi^—P1—O7^iv^	71.14 (14)
O5—Mo2—O4	86.5 (3)	O7—P1—O7^iv^	108.86 (14)
O2—Mo2—O7	160.55 (15)	O7^v^—P1—O7^iv^	108.86 (14)
O50—Mo2—O7	56.0 (2)	O7^iii^—P1—O7^iv^	71.14 (14)
			
O4—O40—Mo1—O1	−21.8 (14)	O1—Mo1—O7—O8	111.4 (4)
Mo2—O40—Mo1—O1	−131.3 (6)	O40—Mo1—O7—O8	52.7 (4)
O4—O40—Mo1—O50^iii^	162.3 (11)	O50^iii^—Mo1—O7—O8	−112.8 (4)
Mo2—O40—Mo1—O50^iii^	52.7 (9)	O30—Mo1—O7—O8	170.8 (4)
O4—O40—Mo1—O30	91.4 (13)	O30^iii^—Mo1—O7—O8	−23.2 (4)
Mo2—O40—Mo1—O30	−18.2 (7)	O3—Mo1—O7—O8	155.1 (4)
O4—O40—Mo1—O30^iii^	−128.9 (14)	O3^iii^—Mo1—O7—O8	−28.1 (4)
Mo2—O40—Mo1—O30^iii^	121.6 (7)	O4—Mo1—O7—O8	63.7 (4)
O4—O40—Mo1—O3	72.7 (13)	O5^iii^—Mo1—O7—O8	−111.4 (4)
Mo2—O40—Mo1—O3	−36.8 (6)	O7^iii^—Mo1—O7—O8	−69.8 (3)
O4—O40—Mo1—O3^iii^	−116.1 (13)	O1—Mo1—O7—O7^iii^	−178.8 (3)
Mo2—O40—Mo1—O3^iii^	134.4 (6)	O40—Mo1—O7—O7^iii^	122.4 (3)
Mo2—O40—Mo1—O4	−109.5 (17)	O50^iii^—Mo1—O7—O7^iii^	−43.0 (3)
O4—O40—Mo1—O5^iii^	179.8 (10)	O30—Mo1—O7—O7^iii^	−119.4 (3)
Mo2—O40—Mo1—O5^iii^	70.3 (10)	O30^iii^—Mo1—O7—O7^iii^	46.5 (3)
O4—O40—Mo1—O7	139.5 (14)	O3—Mo1—O7—O7^iii^	−135.1 (3)
Mo2—O40—Mo1—O7	30.0 (5)	O3^iii^—Mo1—O7—O7^iii^	41.6 (3)
O4—O40—Mo1—O7^iii^	174.2 (13)	O4—Mo1—O7—O7^iii^	133.5 (3)
Mo2—O40—Mo1—O7^iii^	64.7 (6)	O5^iii^—Mo1—O7—O7^iii^	−41.6 (3)
O3—O30—Mo1—O1	32.5 (10)	O1—Mo1—O7—O7^i^	−109.7 (4)
Mo1^i^—O30—Mo1—O1	128.2 (6)	O40—Mo1—O7—O7^i^	−168.5 (4)
O3—O30—Mo1—O40	−79.8 (9)	O50^iii^—Mo1—O7—O7^i^	26.0 (3)
Mo1^i^—O30—Mo1—O40	16.0 (7)	O30—Mo1—O7—O7^i^	−50.4 (3)
O3—O30—Mo1—O50^iii^	139.3 (10)	O30^iii^—Mo1—O7—O7^i^	115.6 (3)
Mo1^i^—O30—Mo1—O50^iii^	−125.0 (7)	O3—Mo1—O7—O7^i^	−66.0 (3)
O3—O30—Mo1—O30^iii^	−149.9 (9)	O3^iii^—Mo1—O7—O7^i^	110.7 (3)
Mo1^i^—O30—Mo1—O30^iii^	−54.2 (6)	O4—Mo1—O7—O7^i^	−157.4 (3)
Mo1^i^—O30—Mo1—O3	95.7 (11)	O5^iii^—Mo1—O7—O7^i^	27.5 (3)
O3—O30—Mo1—O3^iii^	−172.2 (5)	O7^iii^—Mo1—O7—O7^i^	69.1 (4)
Mo1^i^—O30—Mo1—O3^iii^	−76.5 (8)	O1—Mo1—O7—Mo2	46.0 (4)
O3—O30—Mo1—O4	−63.5 (9)	O40—Mo1—O7—Mo2	−12.7 (3)
Mo1^i^—O30—Mo1—O4	32.3 (7)	O50^iii^—Mo1—O7—Mo2	−178.2 (2)
O3—O30—Mo1—O5^iii^	124.9 (9)	O30—Mo1—O7—Mo2	105.4 (2)
Mo1^i^—O30—Mo1—O5^iii^	−139.4 (6)	O30^iii^—Mo1—O7—Mo2	−88.63 (19)
O3—O30—Mo1—O7	−128.6 (10)	O3—Mo1—O7—Mo2	89.7 (2)
Mo1^i^—O30—Mo1—O7	−32.8 (5)	O3^iii^—Mo1—O7—Mo2	−93.52 (19)
O3—O30—Mo1—O7^iii^	−164.7 (9)	O4—Mo1—O7—Mo2	−1.6 (2)
Mo1^i^—O30—Mo1—O7^iii^	−69.0 (6)	O5^iii^—Mo1—O7—Mo2	−176.75 (17)
O5—O50—Mo2—O2	−18.4 (9)	O7^iii^—Mo1—O7—Mo2	−135.2 (2)
Mo1^i^—O50—Mo2—O2	−133.3 (7)	O1—Mo1—O7—Mo1^i^	−46.5 (4)
O5—O50—Mo2—O6	−117.7 (9)	O40—Mo1—O7—Mo1^i^	−105.3 (3)
Mo1^i^—O50—Mo2—O6	127.3 (7)	O50^iii^—Mo1—O7—Mo1^i^	89.3 (2)
O5—O50—Mo2—O40	93.3 (9)	O30—Mo1—O7—Mo1^i^	12.9 (2)
Mo1^i^—O50—Mo2—O40	−21.6 (7)	O30^iii^—Mo1—O7—Mo1^i^	178.8 (2)
O5—O50—Mo2—O6^iv^	166.0 (7)	O3—Mo1—O7—Mo1^i^	−2.81 (18)
Mo1^i^—O50—Mo2—O6^iv^	51.1 (9)	O3^iii^—Mo1—O7—Mo1^i^	173.92 (18)
Mo1^i^—O50—Mo2—O5	−114.9 (13)	O4—Mo1—O7—Mo1^i^	−94.2 (2)
O5—O50—Mo2—O4	76.3 (9)	O5^iii^—Mo1—O7—Mo1^i^	90.70 (19)
Mo1^i^—O50—Mo2—O4	−38.7 (7)	O7^iii^—Mo1—O7—Mo1^i^	132.3 (3)
O5—O50—Mo2—O7	144.4 (9)	O7^iii^—O7—O8—P1	44.36 (15)
Mo1^i^—O50—Mo2—O7	29.4 (6)	O7^i^—O7—O8—P1	−44.36 (15)
O5—O50—Mo2—O8	177.7 (9)	Mo2—O7—O8—P1	−179.77 (14)
Mo1^i^—O50—Mo2—O8	62.7 (7)	Mo1—O7—O8—P1	105.6 (3)
O4—O40—Mo2—O2	19.0 (13)	Mo1^i^—O7—O8—P1	−104.8 (3)
Mo1—O40—Mo2—O2	132.1 (6)	P1—O7—O8—O7^v^	45.85 (16)
O4—O40—Mo2—O50	−94.2 (12)	O7^iii^—O7—O8—O7^v^	90.21 (18)
Mo1—O40—Mo2—O50	18.9 (6)	O7^i^—O7—O8—O7^v^	1.5 (3)
O4—O40—Mo2—O6	−171.7 (10)	Mo2—O7—O8—O7^v^	−133.9 (2)
Mo1—O40—Mo2—O6	−58.6 (9)	Mo1—O7—O8—O7^v^	151.4 (2)
O4—O40—Mo2—O6^iv^	117.9 (13)	Mo1^i^—O7—O8—O7^v^	−59.0 (5)
Mo1—O40—Mo2—O6^iv^	−129.0 (6)	P1—O7—O8—O7^iv^	−45.85 (16)
O4—O40—Mo2—O5	−75.0 (12)	O7^iii^—O7—O8—O7^iv^	−1.5 (3)
Mo1—O40—Mo2—O5	38.1 (6)	O7^i^—O7—O8—O7^iv^	−90.21 (18)
Mo1—O40—Mo2—O4	113.1 (16)	Mo2—O7—O8—O7^iv^	134.4 (2)
O4—O40—Mo2—O7	−143.5 (13)	Mo1—O7—O8—O7^iv^	59.7 (5)
Mo1—O40—Mo2—O7	−30.3 (5)	Mo1^i^—O7—O8—O7^iv^	−150.7 (2)
O4—O40—Mo2—O8	−177.8 (13)	P1—O7—O8—Mo2	179.77 (14)
Mo1—O40—Mo2—O8	−64.7 (6)	O7^iii^—O7—O8—Mo2	−135.9 (2)
C1^ii^—C2—N1—C1	57.4 (4)	O7^i^—O7—O8—Mo2	135.4 (2)
C2^ii^—C1—N1—C2	−57.3 (4)	Mo1—O7—O8—Mo2	−74.6 (3)
Mo1^i^—O30—O3—Mo1	−147.5 (4)	Mo1^i^—O7—O8—Mo2	75.0 (3)
Mo1—O30—O3—Mo1^i^	147.5 (4)	P1—O7—O8—Mo2^v^	108.0 (3)
O1—Mo1—O3—O30	−149.7 (9)	O7^iii^—O7—O8—Mo2^v^	152.3 (3)
O40—Mo1—O3—O30	100.7 (9)	O7^i^—O7—O8—Mo2^v^	63.6 (4)
O50^iii^—Mo1—O3—O30	−39.1 (9)	Mo2—O7—O8—Mo2^v^	−71.8 (3)
O30^iii^—Mo1—O3—O30	48.1 (14)	Mo1—O7—O8—Mo2^v^	−146.5 (2)
O3^iii^—Mo1—O3—O30	23.6 (10)	Mo1^i^—O7—O8—Mo2^v^	3.1 (6)
O4—Mo1—O3—O30	116.3 (9)	P1—O7—O8—Mo2^iv^	−108.1 (3)
O5^iii^—Mo1—O3—O30	−55.6 (9)	O7^iii^—O7—O8—Mo2^iv^	−63.8 (4)
O7—Mo1—O3—O30	44.5 (9)	O7^i^—O7—O8—Mo2^iv^	−152.5 (3)
O7^iii^—Mo1—O3—O30	15.5 (9)	Mo2—O7—O8—Mo2^iv^	72.1 (3)
O1—Mo1—O3—Mo1^i^	170.2 (3)	Mo1—O7—O8—Mo2^iv^	−2.5 (6)
O40—Mo1—O3—Mo1^i^	60.7 (4)	Mo1^i^—O7—O8—Mo2^iv^	147.1 (2)
O50^iii^—Mo1—O3—Mo1^i^	−79.2 (4)	O2—Mo2—O8—P1	−179.9 (2)
O30—Mo1—O3—Mo1^i^	−40.0 (7)	O50—Mo2—O8—P1	−43.38 (17)
O30^iii^—Mo1—O3—Mo1^i^	8.1 (9)	O6—Mo2—O8—P1	−129.43 (12)
O3^iii^—Mo1—O3—Mo1^i^	−16.5 (4)	O40—Mo2—O8—P1	47.0 (2)
O4—Mo1—O3—Mo1^i^	76.3 (4)	O6^iv^—Mo2—O8—P1	129.51 (12)
O5^iii^—Mo1—O3—Mo1^i^	−95.6 (4)	O5—Mo2—O8—P1	−42.59 (17)
O7—Mo1—O3—Mo1^i^	4.4 (3)	O4—Mo2—O8—P1	46.4 (2)
O7^iii^—Mo1—O3—Mo1^i^	−24.5 (4)	O7—Mo2—O8—P1	0.23 (14)
Mo1—O40—O4—Mo2	−147.5 (7)	O2—Mo2—O8—O7^v^	−107.8 (4)
Mo2—O40—O4—Mo1	147.5 (7)	O50—Mo2—O8—O7^v^	28.7 (4)
O2—Mo2—O4—O40	−162.2 (12)	O6—Mo2—O8—O7^v^	−57.4 (3)
O50—Mo2—O4—O40	85.9 (13)	O40—Mo2—O8—O7^v^	119.1 (4)
O6—Mo2—O4—O40	17 (2)	O6^iv^—Mo2—O8—O7^v^	−158.4 (4)
O6^iv^—Mo2—O4—O40	−60.8 (12)	O5—Mo2—O8—O7^v^	29.5 (4)
O5—Mo2—O4—O40	104.6 (13)	O4—Mo2—O8—O7^v^	118.4 (4)
O7—Mo2—O4—O40	32.1 (12)	O7—Mo2—O8—O7^v^	72.3 (4)
O8—Mo2—O4—O40	2.2 (13)	O2—Mo2—O8—O7^iv^	107.9 (4)
O2—Mo2—O4—Mo1	163.3 (4)	O50—Mo2—O8—O7^iv^	−115.6 (4)
O50—Mo2—O4—Mo1	51.3 (4)	O6—Mo2—O8—O7^iv^	158.3 (4)
O6—Mo2—O4—Mo1	−17.6 (11)	O40—Mo2—O8—O7^iv^	−25.2 (4)
O40—Mo2—O4—Mo1	−34.6 (10)	O6^iv^—Mo2—O8—O7^iv^	57.3 (3)
O6^iv^—Mo2—O4—Mo1	−95.3 (4)	O5—Mo2—O8—O7^iv^	−114.8 (4)
O5—Mo2—O4—Mo1	70.1 (4)	O4—Mo2—O8—O7^iv^	−25.9 (4)
O7—Mo2—O4—Mo1	−2.5 (3)	O7—Mo2—O8—O7^iv^	−72.0 (4)
O8—Mo2—O4—Mo1	−32.4 (4)	O2—Mo2—O8—O7	179.9 (3)
O1—Mo1—O4—O40	159.5 (13)	O50—Mo2—O8—O7	−43.6 (2)
O50^iii^—Mo1—O4—O40	−27.6 (17)	O6—Mo2—O8—O7	−129.66 (18)
O30—Mo1—O4—O40	−89.6 (13)	O40—Mo2—O8—O7	46.8 (2)
O30^iii^—Mo1—O4—O40	48.9 (13)	O6^iv^—Mo2—O8—O7	129.28 (18)
O3—Mo1—O4—O40	−106.6 (13)	O5—Mo2—O8—O7	−42.8 (2)
O3^iii^—Mo1—O4—O40	64.4 (13)	O4—Mo2—O8—O7	46.1 (2)
O5^iii^—Mo1—O4—O40	−1(3)	O2—Mo2—O8—Mo2^v^	−45.7 (3)
O7—Mo1—O4—O40	−35.7 (13)	O50—Mo2—O8—Mo2^v^	90.84 (19)
O7^iii^—Mo1—O4—O40	−5.9 (13)	O6—Mo2—O8—Mo2^v^	4.78 (12)
O1—Mo1—O4—Mo2	−162.3 (4)	O40—Mo2—O8—Mo2^v^	−178.8 (2)
O40—Mo1—O4—Mo2	38.2 (11)	O6^iv^—Mo2—O8—Mo2^v^	−96.28 (18)
O50^iii^—Mo1—O4—Mo2	10.6 (9)	O5—Mo2—O8—Mo2^v^	91.6 (2)
O30—Mo1—O4—Mo2	−51.4 (4)	O4—Mo2—O8—Mo2^v^	−179.4 (2)
O30^iii^—Mo1—O4—Mo2	87.0 (4)	O7—Mo2—O8—Mo2^v^	134.44 (16)
O3—Mo1—O4—Mo2	−68.4 (4)	O2—Mo2—O8—Mo2^iv^	45.9 (3)
O3^iii^—Mo1—O4—Mo2	102.5 (4)	O50—Mo2—O8—Mo2^iv^	−177.6 (2)
O5^iii^—Mo1—O4—Mo2	37.6 (19)	O6—Mo2—O8—Mo2^iv^	96.35 (18)
O7—Mo1—O4—Mo2	2.5 (3)	O40—Mo2—O8—Mo2^iv^	−87.2 (2)
O7^iii^—Mo1—O4—Mo2	32.3 (4)	O6^iv^—Mo2—O8—Mo2^iv^	−4.70 (12)
Mo1^i^—O50—O5—Mo2	153.9 (5)	O5—Mo2—O8—Mo2^iv^	−176.80 (18)
Mo2—O50—O5—Mo1^i^	−153.9 (5)	O4—Mo2—O8—Mo2^iv^	−87.8 (2)
O2—Mo2—O5—O50	163.0 (9)	O7—Mo2—O8—Mo2^iv^	−133.99 (16)
O6—Mo2—O5—O50	61.8 (9)	O7^v^—O8—P1—O8^vi^	0(100)
O40—Mo2—O5—O50	−86.8 (9)	O7^iv^—O8—P1—O8^vi^	0(23)
O6^iv^—Mo2—O5—O50	−30.3 (14)	O7—O8—P1—O8^vi^	0(100)
O4—Mo2—O5—O50	−103.3 (9)	Mo2—O8—P1—O8^vi^	0(100)
O7—Mo2—O5—O50	−30.5 (8)	Mo2^v^—O8—P1—O8^vi^	0(100)
O8—Mo2—O5—O50	−2.4 (9)	Mo2^iv^—O8—P1—O8^vi^	0(23)
O2—Mo2—O5—Mo1^i^	−169.1 (3)	O7^v^—O8—P1—O7^i^	−60.0
O50—Mo2—O5—Mo1^i^	27.9 (7)	O7^iv^—O8—P1—O7^i^	180.0
O6—Mo2—O5—Mo1^i^	89.7 (3)	O7—O8—P1—O7^i^	60.0
O40—Mo2—O5—Mo1^i^	−58.9 (4)	Mo2—O8—P1—O7^i^	59.75 (15)
O6^iv^—Mo2—O5—Mo1^i^	−2.4 (9)	Mo2^v^—O8—P1—O7^i^	−60.25 (15)
O4—Mo2—O5—Mo1^i^	−75.4 (3)	Mo2^iv^—O8—P1—O7^i^	179.75 (15)
O7—Mo2—O5—Mo1^i^	−2.6 (3)	O7^v^—O8—P1—O7^vi^	60.0
O8—Mo2—O5—Mo1^i^	25.5 (3)	O7^iv^—O8—P1—O7^vi^	−60.0
O2—Mo2—O6—Mo2^v^	153.3 (3)	O7—O8—P1—O7^vi^	180.0
O50—Mo2—O6—Mo2^v^	−95.6 (4)	Mo2—O8—P1—O7^vi^	179.75 (15)
O40—Mo2—O6—Mo2^v^	−16.4 (6)	Mo2^v^—O8—P1—O7^vi^	59.75 (15)
O6^iv^—Mo2—O6—Mo2^v^	52.1 (4)	Mo2^iv^—O8—P1—O7^vi^	−60.25 (15)
O5—Mo2—O6—Mo2^v^	−112.6 (3)	O7^v^—O8—P1—O7	−120.0
O4—Mo2—O6—Mo2^v^	−25.8 (10)	O7^iv^—O8—P1—O7	120.0
O7—Mo2—O6—Mo2^v^	−40.2 (3)	Mo2—O8—P1—O7	−0.25 (15)
O8—Mo2—O6—Mo2^v^	−9.7 (3)	Mo2^v^—O8—P1—O7	−120.25 (15)
O2—Mo2—O7—P1	179.9 (3)	Mo2^iv^—O8—P1—O7	119.75 (15)
O50—Mo2—O7—P1	124.1 (3)	O7^iv^—O8—P1—O7^v^	−120.0
O6—Mo2—O7—P1	43.7 (3)	O7—O8—P1—O7^v^	120.0
O40—Mo2—O7—P1	−121.9 (4)	Mo2—O8—P1—O7^v^	119.75 (15)
O6^iv^—Mo2—O7—P1	−44.2 (3)	Mo2^v^—O8—P1—O7^v^	−0.25 (15)
O5—Mo2—O7—P1	135.7 (3)	Mo2^iv^—O8—P1—O7^v^	−120.25 (15)
O4—Mo2—O7—P1	−132.4 (3)	O7^v^—O8—P1—O7^iii^	180.0
O8—Mo2—O7—P1	−0.22 (13)	O7^iv^—O8—P1—O7^iii^	60.0
O2—Mo2—O7—O8	−179.9 (3)	O7—O8—P1—O7^iii^	−60.0
O50—Mo2—O7—O8	124.3 (3)	Mo2—O8—P1—O7^iii^	−60.25 (15)
O6—Mo2—O7—O8	43.93 (13)	Mo2^v^—O8—P1—O7^iii^	179.75 (15)
O40—Mo2—O7—O8	−121.6 (3)	Mo2^iv^—O8—P1—O7^iii^	59.75 (15)
O6^iv^—Mo2—O7—O8	−44.02 (13)	O7^v^—O8—P1—O7^iv^	120.0
O5—Mo2—O7—O8	135.9 (2)	O7—O8—P1—O7^iv^	−120.0
O4—Mo2—O7—O8	−132.2 (2)	Mo2—O8—P1—O7^iv^	−120.25 (15)
O2—Mo2—O7—O7^iii^	−109.2 (3)	Mo2^v^—O8—P1—O7^iv^	119.75 (15)
O50—Mo2—O7—O7^iii^	−165.0 (4)	Mo2^iv^—O8—P1—O7^iv^	−0.25 (15)
O6—Mo2—O7—O7^iii^	114.6 (2)	O8—O7—P1—O8^vi^	180.0
O40—Mo2—O7—O7^iii^	−50.9 (3)	O7^iii^—O7—P1—O8^vi^	59.27 (17)
O6^iv^—Mo2—O7—O7^iii^	26.7 (2)	O7^i^—O7—P1—O8^vi^	−59.27 (17)
O5—Mo2—O7—O7^iii^	−153.4 (3)	Mo2—O7—P1—O8^vi^	−179.74 (15)
O4—Mo2—O7—O7^iii^	−61.5 (3)	Mo1—O7—P1—O8^vi^	59.6 (2)
O8—Mo2—O7—O7^iii^	70.69 (16)	Mo1^i^—O7—P1—O8^vi^	−58.9 (2)
O2—Mo2—O7—O7^i^	109.2 (3)	O7^iii^—O7—P1—O8	−120.73 (17)
O50—Mo2—O7—O7^i^	53.3 (3)	O7^i^—O7—P1—O8	120.73 (17)
O6—Mo2—O7—O7^i^	−27.0 (2)	Mo2—O7—P1—O8	0.26 (15)
O40—Mo2—O7—O7^i^	167.4 (4)	Mo1—O7—P1—O8	−120.4 (2)
O6^iv^—Mo2—O7—O7^i^	−115.0 (2)	Mo1^i^—O7—P1—O8	121.1 (2)
O5—Mo2—O7—O7^i^	65.0 (3)	O8—O7—P1—O7^i^	−120.73 (17)
O4—Mo2—O7—O7^i^	156.8 (3)	O7^iii^—O7—P1—O7^i^	118.5 (3)
O8—Mo2—O7—O7^i^	−70.97 (16)	Mo2—O7—P1—O7^i^	−120.5 (2)
O2—Mo2—O7—Mo1	−46.0 (4)	Mo1—O7—P1—O7^i^	118.9 (4)
O50—Mo2—O7—Mo1	−101.8 (2)	Mo1^i^—O7—P1—O7^i^	0.36 (16)
O6—Mo2—O7—Mo1	177.83 (12)	O8—O7—P1—O7^vi^	60.87 (7)
O40—Mo2—O7—Mo1	12.3 (2)	O7^iii^—O7—P1—O7^vi^	−59.9 (2)
O6^iv^—Mo2—O7—Mo1	89.88 (12)	O7^i^—O7—P1—O7^vi^	−178.39 (15)
O5—Mo2—O7—Mo1	−90.2 (2)	Mo2—O7—P1—O7^vi^	61.1 (2)
O4—Mo2—O7—Mo1	1.7 (2)	Mo1—O7—P1—O7^vi^	−59.5 (2)
O8—Mo2—O7—Mo1	133.89 (18)	Mo1^i^—O7—P1—O7^vi^	−178.03 (18)
O2—Mo2—O7—Mo1^i^	46.0 (4)	O8—O7—P1—O7^v^	−59.27 (17)
O50—Mo2—O7—Mo1^i^	−9.9 (2)	O7^iii^—O7—P1—O7^v^	180.0
O6—Mo2—O7—Mo1^i^	−90.25 (12)	O7^i^—O7—P1—O7^v^	61.5 (3)
O40—Mo2—O7—Mo1^i^	104.2 (3)	Mo2—O7—P1—O7^v^	−59.0 (2)
O6^iv^—Mo2—O7—Mo1^i^	−178.20 (12)	Mo1—O7—P1—O7^v^	−179.64 (15)
O5—Mo2—O7—Mo1^i^	1.74 (18)	Mo1^i^—O7—P1—O7^v^	61.8 (4)
O4—Mo2—O7—Mo1^i^	93.6 (2)	O8—O7—P1—O7^iii^	120.73 (17)
O8—Mo2—O7—Mo1^i^	−134.18 (18)	O7^i^—O7—P1—O7^iii^	−118.5 (3)
O1—Mo1—O7—P1	−179.1 (2)	Mo2—O7—P1—O7^iii^	121.0 (2)
O40—Mo1—O7—P1	122.1 (4)	Mo1—O7—P1—O7^iii^	0.36 (15)
O50^iii^—Mo1—O7—P1	−43.3 (3)	Mo1^i^—O7—P1—O7^iii^	−118.2 (4)
O30—Mo1—O7—P1	−119.7 (3)	O8—O7—P1—O7^iv^	59.27 (17)
O30^iii^—Mo1—O7—P1	46.2 (3)	O7^iii^—O7—P1—O7^iv^	−61.5 (3)
O3—Mo1—O7—P1	−135.4 (3)	O7^i^—O7—P1—O7^iv^	180.0
O3^iii^—Mo1—O7—P1	41.3 (3)	Mo2—O7—P1—O7^iv^	59.5 (2)
O4—Mo1—O7—P1	133.2 (3)	Mo1—O7—P1—O7^iv^	−61.1 (4)
O5^iii^—Mo1—O7—P1	−41.9 (3)	Mo1^i^—O7—P1—O7^iv^	−179.64 (16)
O7^iii^—Mo1—O7—P1	−0.32 (13)		

Symmetry codes: (i) *y*, −*x*+*y*, −*z*; (ii) −*x*+2/3, −*y*+1/3, −*z*+1/3; (iii) *x*−*y*, *x*, −*z*; (iv) −*y*, *x*−*y*, *z*; (v) −*x*+*y*, −*x*, *z*; (vi) −*x*, −*y*, −*z*.

### Hydrogen-bond geometry (Å, °)

**Table:**

*D*—H···*A*	*D*—H	H···*A*	*D*···*A*	*D*—H···*A*
N1—H1C···O6	0.90	2.22	2.812 (4)	123
N1—H1D···O50	0.90	2.43	2.926 (6)	115
N1—H1C···O4^v^	0.90	2.20	3.041 (7)	155
N1—H1C···O40^v^	0.90	2.16	3.047 (7)	168
N1—H1C···O30^i^	0.90	2.52	3.091 (6)	122
N1—H1D···O5	0.90	2.19	2.852 (6)	130
N1—H1D···O1^vii^	0.90	2.14	2.900 (4)	142
C1—H1A···O6	0.97	2.58	3.101 (5)	114
C1—H1B···O4^viii^	0.97	2.58	3.347 (7)	137
C2—H2A···O2^viii^	0.97	2.43	3.291 (4)	148
C2—H2B···O3^i^	0.97	2.43	3.156 (6)	132
C2—H2B···O2^ix^	0.97	2.42	3.068 (4)	124

Symmetry codes: (v) −*x*+*y*, −*x*, *z*; (i) *y*, −*x*+*y*, −*z*; (vii) *x*+1/3, *x*−*y*+2/3, *z*+1/6; (viii) *x*−*y*+2/3, *x*+1/3, −*z*+1/3; (ix) −*y*+2/3, −*x*+1/3, *z*−1/6.

## References

[bb1] Brandenburg, K. (1999). *DIAMOND* Crystal Impact GbR, Bonn, Germany.

[bb2] Brown, I. D. & Altermatt, D. (1985). *Acta Cryst.* B**41**, 244–247.

[bb3] Han, Z. G., Zhao, Y. L., Peng, J., Tian, A. X., Liu, Q., Ma, J. F., Wang, E. B. & Hu, N. H. (2005). *CrystEngComm*, **7**, 380–387.

[bb4] Higashi, T. (1995). *ABSCOR* Rigaku Corporation, Tokyo, Japan.

[bb5] Hill, C. L. (1998). *Chem. Rev.* **98**, 1–2. 10.1021/cr960395y 11851497

[bb6] Kurth, D. G., Volkmer, D., Pope, M. T. & Müller, A. (2001). *Polyoxometalate Chemistry*, p. 301. Dordrecht: Kluwer.

[bb7] Li, Y. G., Dai, L. M., Wang, Y. H., Wang, X. L., Wang, E. B., Su, Z. M. & Xu, L. (2007). *Chem. Commun.* pp. 2593–2595. 10.1039/b700511c 17579748

[bb8] Pope, M. T. & Müller, A. (1991). *Angew. Chem. Int. Ed. Engl.* **30**, 34–48.

[bb9] Rigaku (1998). *PROCESS-AUTO.* Rigaku Corporation, Tokyo, Japan.

[bb10] Sheldrick, G. M. (2008). *Acta Cryst.* A**64**, 112–122. 10.1107/S0108767307043930 18156677

[bb11] Yuan, J.-H., Wang, C., Yu, M.-J. & Li, J. (2008). *Acta Cryst.* E**64**, m831. 10.1107/S1600536808014463 PMC2961430 21202512

